# Management of Rheumatoid Arthritis: An Overview

**DOI:** 10.3390/cells10112857

**Published:** 2021-10-23

**Authors:** Andrei-Flavius Radu, Simona Gabriela Bungau

**Affiliations:** 1 Doctoral School of Biological and Biomedical Sciences, University of Oradea, 410087 Oradea, Romania; 2 Department of Pharmacy, Faculty of Medicine and Pharmacy, University of Oradea, 410028 Oradea, Romania

**Keywords:** rheumatoid arthritis, anti-citrullinated protein antibodies, rheumatoid factor, extra-articular manifestations, DMARDs, Janus kinase inhibitors, targets, proteins

## Abstract

Rheumatoid arthritis (RA) is a multifactorial autoimmune disease of unknown etiology, primarily affecting the joints, then extra-articular manifestations can occur. Due to its complexity, which is based on an incompletely elucidated pathophysiological mechanism, good RA management requires a multidisciplinary approach. The clinical status of RA patients has improved in recent years due to medical advances in diagnosis and treatment, that have made it possible to reduce disease activity and prevent systemic complications. The most promising results were obtained by developing disease-modifying anti-rheumatic drugs (DMARDs), the class to which conventional synthetic, biologic, and targeted synthetic drugs belong. Furthermore, ongoing drug development has led to obtaining molecules with improved efficacy and safety profiles, but further research is needed until RA turns into a curable pathology. In the present work, we offer a comprehensive perspective on the management of RA, by centralizing the existing data provided by significant literature, emphasizing the importance of an early and accurate diagnosis associated with optimal personalized treatment in order to achieve better outcomes for RA patients. In addition, this study suggests future research perspectives in the treatment of RA that could lead to higher efficacy and safety profiles and lower financial costs.

## 1. Introduction

Rheumatoid arthritis (RA) is defined as a systemic autoimmune pathology associated with a chronic inflammatory process, which can damage both joints and extra-articular organs, including the heart, kidney, lung, digestive system, eye, skin and nervous system [1,2]. Numerous types of arthritis have been investigated and described in order to classify them into non-inflammatory arthritis (osteoarthritis) and inflammatory arthritis caused by crystal deposition (pseudogout, basic calcium phosphate disease, gout), by bacterial and viral infections (*Staphylococcus aureus*, *Neisseria gonorrhea*, complications of Lyme disease, *Parvovirus*, *Enterovirus*) or by autoimmune processes.

The heterogeneous group of autoimmune rheumatic diseases also includes systemic lupus erythematosus (SLE), Sjögren’s syndrome, adult-onset scleroderma, spondylarthritis (SpA), psoriatic arthritis (PsA), polymyositis (PM), etc. Due to the fact that they may be similar in signs and symptoms, differential diagnosis is essential [3].

Although a number of biomolecular mechanisms have been proposed, the etiology of RA is not yet fully elucidated, a current hypothesis being that dysregulated citrullination leads to the production of anti-citrullinated protein antibodies (ACPAs) [4,5]. The evolution of RA is fluctuant with episodic exacerbations and in the absence of optimal treatment symptoms gradually worsen until the joints are irreversibly damaged and physical and psychological functioning is affected [6]. Moreover, RA complications and comorbidities reduce the life expectancy of patients by a few years [7].

Existing statistical analysis and interpretation of quantitative data show that RA represents not only a medical feature, but also a public health issue. The most common medical cause of mobility-related functionality loss among United States (US) adults is arthritis [8,9]. Furthermore, several health economic studies have measured the economic burden of RA and, and as a result, have demonstrated that the costs of preventing RA by reducing the risk factors or treating incipient cases are much lower than those generated by hospitalization and surgeries [10,11].

Due to major advances in the pharmaceutical industry, new therapeutic approaches are available. However, the lack of understanding of the molecular mechanisms governing the fate of antibodies leads to a challenge in order to discover a curative treatment. The most effective therapeutic approach requires early diagnosis and an optimal nonpharmacological and pharmacological treatment, associated with periodic evaluation of therapeutic efficacy and safety. The target of therapy is to obtain remission and to reduce side effects [12]. Pharmacological agents that help maintain joint function can be classified as conventional synthetic disease-modifying antirheumatic drugs (DMARDs), biologic DMARDs and targeted synthetic DMARDs, which are included in a new class of nonbiologic DMARDs by the American College of Rheumatology (ACR) [13]. Inadequate symptom control in RA patients requires the use of nonsteroidal anti-inflammatory drugs (NSAIDs) and glucocorticoids (GCs) as adjunctive therapy in reducing inflammation [14].

This review summarizes and filters scholarly publications on RA between 1987 and 2021 provided by a systematic literature search related to epidemiological data, diagnostic, prognostic and predictive biomarkers, pathophysiological mechanisms, prevention strategies, nonpharmacological and pharmacological approaches, with an emphasis on new biological therapies. Moreover, it presents in detail the safety and the efficacy profile of the pharmacological agents approved by the US Food and Drug Administration (FDA) and European Medicines Agency (EMA). In this regard, literature research was conducted by searching some of the most well-known scientific databases (i.e., MDPI, Medline, Embase, ScienceDirect, Google Scholar, Web of Science, Scopus, Access Pharmacy, etc.). Furthermore, they were used with two controlled vocabulary thesauri. Medical subject heading terms (MeSH) were used for searching in PubMed, and Embase subject headings (Emtree) for searching in Embase (i.e., “autoimmune rheumatic diseases”, “rheumatoid arthritis”, “epidemiology of rheumatoid arthritis”, “diagnostic, prognostic and theranostic of rheumatoid arthritis”, “pathophysiology of rheumatoid arthritis”, “therapeutic management of rheumatoid arthritis”, “conventional DMARDs”, “biologic DMARDs”, “targeted synthetic DMARDs”, “current and future trends of rheumatoid arthritis“). A total of 240 bibliographic references were selected and cited to validate the information in this review.

The present research aims to provide a comprehensive overview of RA, centralizing updated information regarding recent advances in diagnosis and therapeutic approaches, to support specialists and patients to improve the management of RA. Moreover, emphasizing the efficacy and safety profiles of new biologic DMARD’s can help specialists decide to switch from conventional DMARD’s to targeted therapy, also providing updated information for rheumatology guidelines. In addition, relevant scientific information was systematically evaluated, and the focus was on optimizing the management of RA, both by examining evidence-based medicine articles and by updating and centralizing the new therapeutic approaches with the implementation of personalized medicine in a context of an incurable disease.

## 2. Epidemiologic Overview

During the last 30 years numerous scientists have extensively studied variation of the prevalence and incidence of RA. These studies have demonstrated that RA is a global disease distributed worldwide, regardless of race, sex, ethnicity, nationality, age, etc. However, the results of prevalence and incidence measurements vary depending on the population characteristics and have changed over time [15].

### 2.1. Prevalence of RA in Epidemiological Studies

Epidemiological studies measuring the prevalence of RA in a few European, Asian, North American, and South American countries between 1990 and 2005 reported pertinent and relevant results. Low prevalence ratios were reported in Serbia (0.18%) [16], China (0.28%) [17], France (0.31%) [18], Italy (0.33%) [19], and the US (0.41%) [17], while higher prevalence ratios were observed in Japan (1.7%) [20] and Argentina (1.97%) [21]. It is worth pointing out that older studies can face methodological biases resulting in differences in the prevalence of RA because the types of studies conducted were significantly different: cross-sectional studies, random selection, telephone survey, postal questionnaire, inception cohort, outpatient, and hospitalization medical records. Moreover, it was observed that gender differences exist in the prevalence of RA. All the studies reported a three- to five-fold higher prevalence of RA in females than males. The most significant difference was reported by the Argentinian study (women 3.2%, men 0.6%), while the closest values were reported in Serbia (women 0.29%, men 0.09%) [17].

Trends in the prevalence of RA have been assessed over the years, and the results are presented in Table 1.

The prevalence of RA has been rising almost unanimously since 1990 up to date. The largest increase was observed in the Spanish population. However, in Japan and Argentina the prevalence ratios have decreased over the years.

Nowadays, the global prevalence ratio of RA is about 1% and it is more common in women, with small continuous fluctuations and an apparent growth from south to north, and from countryside to metropolitan areas [31].

### 2.2. Incidence of RA in Epidemiological Studies

From an epidemiological perspective, the incidence of RA varies by age and population. Studies have been conducted over years to measure the incidence in certain geographical areas and for identifying variables that have led to different results. The data collection methods used were types of observational studies, including inception cohort, longitudinal population-based study, review of medical records, and prospective case-control studies, and were conducted between 1985 and 2002 [17]. Lower incidence rates have been reported in Japan (8 cases per 100,000 inhabitants) [20], and France (8.8 cases per 100,000 inhabitants) [32]. The highest incidence rate has been observed in the US (44.6 cases per 100,000 inhabitants) [33]. It has also been reported that the incidence in women is significantly higher than in men. However, recent studies have reported a fluctuating incidence over the past three decades. Therefore, the incidence ratios in the US ranged from 40 cases per 100,000 inhabitants in 1994 to 43 cases per 100,000 inhabitants in 2004 and nowadays RA has an incidence of 41 cases per 100,000 inhabitants [34].

The influence of age on the incidence of RA has been assessed by studies that have shown an increase with age up to 80 years when it begins to decline. Moreover, the incidence rate has decreased progressively in the last 60 years, being much more significant among women [17].

Several studies have reported differences in incidence rates at the regional level within countries. One potential explanation for these variations may have been environmental exposure to chemicals, climatic changes, infectious diseases, and food [35,36]. Furthermore, it has been reported that people with a low socio-economic background, living in rural areas during childhood, are at a higher risk of developing RA in adulthood [37]. The latest studies have reported that the United Kingdom has the highest standardized incidence rate (27.5 cases per 100,000 inhabitants) and Canada has had the biggest rise in the incidence rate in the last 30 years [15,38]. The reasons for the increase in the incidence rate have no unequivocal explanation, but risk factors may play an important role.

### 2.3. Risk Factors for RA

RA is a multifactorial disease caused by genetic, environmental and stochastic factors [39]. The genetic risk for RA that has been estimated by scientific studies is about 50% [40,41]. The presence or absence of rheumatoid factor (RF) and ACPAs can divide RA into two types (seropositive and seronegative) and there are also differences between the risk factors involved [42,43]. Tyrosine phosphatase non-receptor type 22 (PTPN22) risk alleles [44,45], human leukocyte antigen D-related (HLA-DR) alleles [42], and tumors necrosis factor-receptor associated factor 1 and complement component 5 (TRAF1/C5) related genes are the main genetic factors associated with an ACPA-positive subtype [46], while interferon regulatory factor 5 (IRF-5) is confined to the ACPA-negative subtype [47].

As significant contributors to population health, environmental risk factors play an important role in the management of RA. Like other diseases, smoking is linked to the development or exacerbation of RA. The first evidence of the association of smokers with an increased risk of RA was observed by serendipity in a study with a different purpose [48]. Since then, it has become the best described risk factor for RA. The harmful chemicals in tobacco products have been comprehensively evaluated and the results suggest that smoking delivers a specific signal. Smoking might be related to a genetic context with a specific role in triggering a particular subtype of RA [49]. It has been reported that smoking affects RF- or ACPA-positive RA [50], and has no or very little effect on ACPA-negative RA [51]. Moreover, the risk of developing ACPA-positive RA is much higher in smokers who carry HLA-DR Beta 1 shared epitope alleles [52]. It has not been observed that any association exists between passive smokers and the risk of developing RA [53].

Exposure to silica dust is an occupational type of exposure that impacts RA. It has been reported there is an association between silicosis and RA, mainly affecting patients with ACPA-positive RA [54]. Chronic exposure to silica can lead to rheumatoid pneumoconiosis, also known as Caplan’s syndrome, a rare disease of RA patients who have developed silicosis [55].

Dietary factors and consuming habits have also been evaluated over time. Dietary agents influence RA and the evidence has shown that fasting periods and vegetarian diets can decrease the evolution of RA. Moreover, avoiding red meat and increasing fruit and oily fish consumption can be associated with a decreased risk for RA [56,57]. Coffee consumption may be a risk factor for RA, a possible explanation being the involvement in the production of RF [58]. It has been reported in a case-control study that alcohol consumption may have a beneficial effect on RA by lowering the risk of developing ACPA-positive RA, but this hypothesis requires additional investigation [49]. Therefore, a personalized diet for each person should be considered.

Infections are biological risk factors that might trigger the development of RA. A comparative cohort study reported that the risk of joint, skin and bone infections is much higher in patients with RA compared with non-inflammatory rheumatic diseases [59]. Moreover, bacterial triggers have also been identified in the case of Lyme arthritis, a pathology with many similarities to RA [60].

*Porphyromonas gingivalis* is a pathogenic bacterium that causes periodontal disease. Due to its role in inducing citrullination and promoting osteoclast genesis, an association between RA and periodontal disease has been reported [61].

A comprehensive characterization of the interaction between environment, genes and stochastic factors may be the basis for understanding the complexity of the biomolecular mechanisms that coordinate RA.

## 3. Pathophysiology of RA

Although the pathophysiological mechanisms for RA are not fully elucidated, several hypotheses have been postulated. It has been reported that immunological processes can occur many years before symptoms of joint inflammation are noticed, the so-called pre-RA phase [62]. The interactions between epigenetic modifications on the genomic structure and environmental factors can lead to modified self-antigens as in the case of immunoglobulin G (IgG), type 2 collagen and vimentin. These proteins with arginine residues can be converted to citrulline by peptidyl arginine deiminases in a post-translational modification called citrullination [63,64]. Moreover, joint disorders like synovial hyperplasia or synovial infections can trigger cytokine release that may cause joint inflammation and also modified self-antigens [65].

Due to the susceptibility genes HLA-DR1 and HLA-DR4, the immune system is no longer able to recognize citrullinated proteins (vimentin, type II collagen, histones, fibrin, fibronectin, Epstein-Barr nuclear antigen 1, α-enolase) as self-structures [66]. Antigens are taken up by antigen-presenting cells (APC), which are dendritic cells that are activated to initiate an immune response. The whole complex migrates to the lymph node, where the activation of CD4^+^ helper T cells takes place. Furthermore, the germinal center of the lymph node contains B cells that get activated by reciprocal and sequential signals with T cells, an immunological process called costimulation.

**Figure 1:**
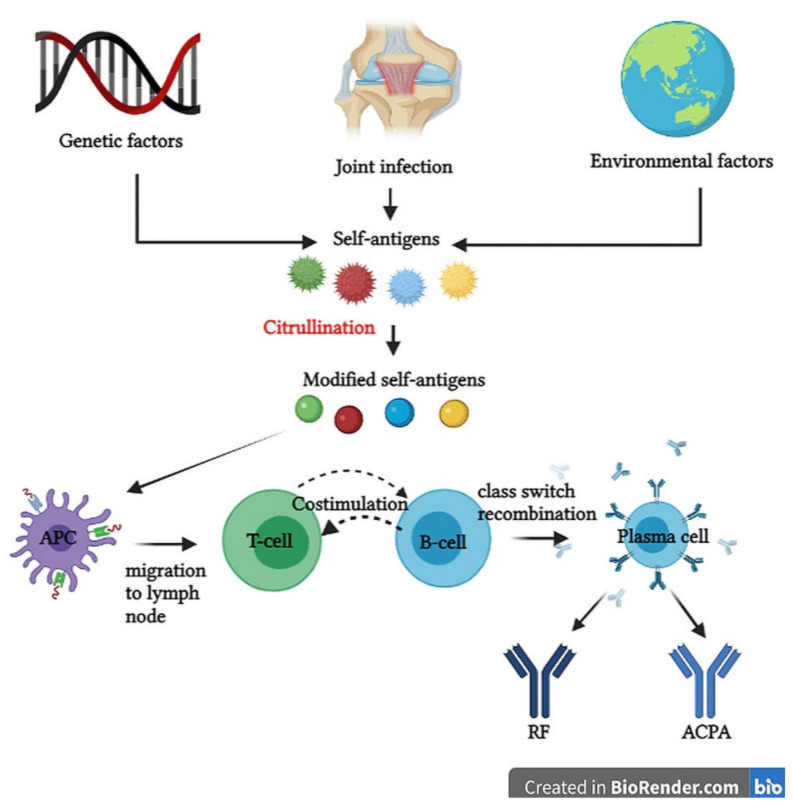
Immunological processes in the pre-RA phase. ACPA, anti-citrullinated protein antibodies; APC, antigen-presenting cells; RF, rheumatoid factor.

An example of costimulation is the interaction between CD28 and CD80/86 [67,68]. At this level, B cells undergo somatic hypermutation or class-switch recombination and start to proliferate and differentiate intro plasma cells that produce autoantibodies depending on the receptors of the precursor cells [69]. Autoantibodies are proteins produced by an immune system that no longer discriminates self from non-self-structures, so self-tissues and organs are accidentally targeted. RF and ACPA are the most studied autoantibodies involved in RA. RF is an IgM antibody with a testing specificity of 85% in RA patients, which targets the Fc portion of IgG, also called the constant region [70]. It also forms an immune complex with IgG and complement protein, a complex able to migrate in the synovial fluid. However, ACPA is more specific for RA and targets citrullinated proteins and after their binding interactions, immune complexes are formed with an accumulation in the synovial fluid [71]. All the features of an immune response in the pre-RA phase are summarized in Figure 1.

In the realm of RA, air pollution, which consists of a mixture of suspended particulate materials (PM) of various sizes and gases (nitrates, ozone, sulfur dioxide and carbon monoxide), has recently received increasing attention. Pollutants are released into the air through a variety of man-made and natural sources, including agriculture, fossil fuel combustion, chemical industries, use of solvents, volcanic eruptions, windblown dust, emissions from plants, etc. The clinical impact of air pollution is primarily considered in relation to respiratory diseases. The alveoli, an important part of the respiratory system that filters oxygen and carbon dioxide, have been reported to be damaged by ozone. Pollutants can also cause secondary harm to lung tissue by reacting with different enzymes, resulting in pulmonary inflammation or infection. Three major epidemiological investigations conducted in the United States, Canada and Sweden have demonstrated that air pollutants can be linked to the pathogenesis of RA [72].

Alsaber et al. (2020), conducted a study to investigate the correlations between air pollutants and RA activity through regression models. Nitrates and sulfur dioxide were discovered to be important risk factors for the development of RA [73].

One of the latest research studies that has been published is a case-crossover study (which assessed a potential association between air pollutants in the Verona area and RA evolution) in 888 RA patients, showed that air pollution is linked to high C-reactive protein levels (CRP), to the severity of RA illness and its reactivations due to a poor response to biological therapies [74].

The involvement of air pollutants in the pathogenesis of RA may be based on a few mechanistic processes. Free reactive oxygen species (ROS) generated by PM inhalation can activate nuclear factor kappa B (NF-KB), which activates T helper cell type 1 (Th1) to produce tumor necrosis factor alpha (TNF-α), interleukin-1 (IL-1) and interleukin-6 (IL-6). These cytokines promote the maturation of resting monocytes into mature dendritic cells, which then offer auto-antigens to self-reactive T lymphocytes, causing them to move to target tissues and promote joint inflammation and erosion. Moreover, the citrullination of arginine amino acid residues into citrullinated peptides is also aided by ROS, which promotes chronic lung disease and systemic inflammation. ACPAs, which are generated by biochemical reactions, trigger an immunological response by binding to cellular Fc receptors and activating complement, resulting in joint inflammation and bone erosion [72].

Reduced ultraviolet B (UVB) radiation causes a decrease in 1,25-dihydroxyvitamin D3 production in the skin, which functions as an immunomodulator by activating the vitamin D receptor (VDR). As a result, the immunomodulatory functions are not optimal, and this can trigger RA [75].

Another important element with major implications in the pathogenesis of RA is the gut microbiota, the most densely colonized bacterial population within the human body [76]. RA etiology is also related to intestinal dysbiosis, which leads to certain autoimmune pathways and mechanisms, such as stimulation of APC by activating toll-like receptors (TLRs) or nod-like receptors (NLRs), molecular mimicry, alterations in intestinal permeability, promotion of T cell differentiation and amplification of mucosal inflammation via certain pathways [77].

It has been demonstrated that immunological, metabolic, and neurobehavioral features are influenced by gut microorganisms. When compared to healthy controls, RA patients showed significant differences in the gut microbiota composition, being associated with an increase or a decrease in certain bacterial populations [78].

The gastrointestinal microbiota can impact the development of RA through proximal intestinal immunomodulatory cells, which are found in specific locations within the gut. Several case-control studies have demonstrated quantitative changes in specific bacteria in RA patients by 16S rRNA sequencing and metagenomic shotgun sequencing. According to the results of the studies, *Prevotella copri*, *Collinsella* and *Lactobacillus salivarius* were found to be more abundant in RA patients, while *Bacteroides*, *Faecalibacterium*, *Veillonella* and *Haemophilus* were lower in quantity [76,79].

**Figure 2:**
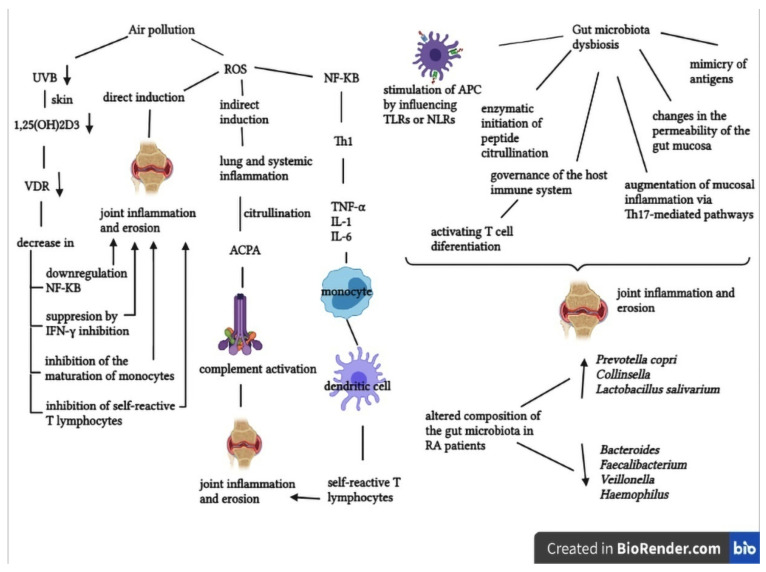
The involvement of air pollution and microbiota in the pathogenesis of RA. ACPA, anti-citrullinated protein antibodies; APC, antigen-presenting cells; IFN, interferon gamma; IL, interleukin; NF-KB, nuclear factor kappa-light-chain-enhancer of activated B cells; NLR, nod-like receptor; RA, rheumatoid arthritis; ROS, reactive oxygen species; TLR, toll-like receptor; TNF-α, tumor necrosis factor alpha; UVB, ultraviolet B radiation; VDR, vitamin D receptor.

The mechanisms underlying the involvement of air pollutants and gut microbiota in the pathogenesis of RA are shown in Figure 2 [72,76,78].

RA is generally characterized by an insidious onset of symptoms, but over time the disease progresses and gradually worsens. The trigger for RA symptoms is unknown, but the immunological processes that take place in the synovium and in the synovial fluid have been described [80]. Synovial macrophages release cytokines like tumor necrosis factor alpha (TNF-α), interleukin-1 (IL-1) and interleukin-6 (IL-6), which are associated with inflammatory processes, stimulation of fibroblast-like synoviocytes (FLS) and stimulation of osteoclast activity [81]. Increased osteoclast activity and maturation leads to bone erosion. Once activated, FLS are specialized cells that can produce matrix metalloproteinase (MMP) [82]. MMP can lead to cartilage degradation and the cartilage also secrets proteases in a feedback mechanism [83,84]. FLS can migrate from joint to joint, creating a pattern of symmetrical RA [12]. Moreover, FLS stimulates receptor activator of nuclear factor-kB ligand (RANKL) expression, allowing T cells to bind proteins on the surface of osteoclasts, which also leads to bone erosion by increasing osteoclast activity [85].

CD$4^{+}$ T cells promote inflammation, bone erosion and cartilage degradation by stimulating RANKL expression and producing interleukin 17 (IL-17), with an important role in the stimulation of synovial macrophages and FLS [86,87]. Plasma cells also promote inflammation through cytokines and autoantibodies [88].

In the synovial fluid the presence of neutrophils has been reported, which produce proteases and reactive oxygen species (ROS) that may cause bone erosion and cartilage degradation [89,90]. Immune complexes have also been identified in the synovial fluid such as antibodies that bind one to another, promote inflammation and over-activate the complement system [91].

**Figure 3:**
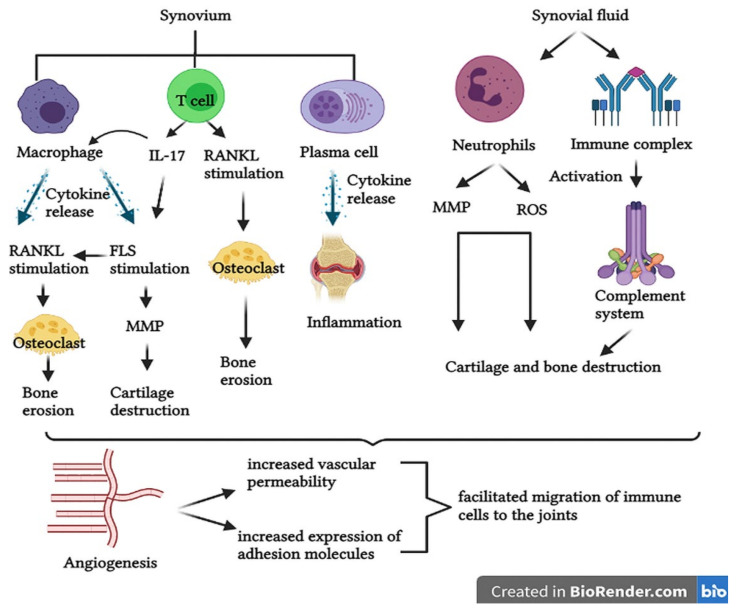
Pathological mechanisms in RA. IL, interleukin; FLS, fibroblast-like synoviocytes; MMP, matrix metalloproteinase; RANKL, receptor activator of nuclear factor-kB ligand; ROS, reactive oxygen species.

Angiogenesis is a process of forming new blood vessels from existing ones, which also occurs in RA. In contrast to its beneficial role in many physiological processes, in RA it plays a critical role because the immune cells can migrate into the joints due to the increase in vascular permeability and the expression of adhesion molecules (vascular adhesion molecule 1) [92,93]. Furthermore, vascular endothelial growth factor (VEGF) is a proangiogenic factor located in the synovium in RA patients, which has a potent role in bone destruction as a promoter of osteoclast genesis [92]. The pathophysiological processes that lead to the appearance of symptoms in RA are summarized in Figure 3.

The complexity of this pathology is also based on numerous signaling molecules with specific roles in inflammatory processes. Janus kinases (JAKs) are small signaling proteins with pathophysiological relevance because they can represent molecular targets for many therapeutic agents [94]. Thus, further research is needed to elucidate all the pathological mechanisms and to optimize future therapies with high safety and efficacy profiles.

## 4. Clinical Aspects of RA

An essential part of RA management is the evaluation of clinical aspects, including signs and symptoms, prognostic laboratory biomarkers, differential diagnosis, complications, and extra-articular manifestations, etc. Early and accurate diagnosis of RA is highly important in order to differentiate between types of arthritis and types of autoimmune disease and to promptly establish the correct treatment and prevent long-term complications [95].

### 4.1. RA Diagnosis

The 2010 American College of Rheumatology (ACR) and European League Against Rheumatism (EULAR) classification criteria for RA evaluates a set of variables, such as risk factors, number and type of joints involved and the duration of symptoms, in order to redefine the focus from late-stage phase management to the early detection of RA [96]. The classification system exposes conditions to which a certain score corresponds and must be re-examined over time:

- 2–10 large joints corresponding to 1;
- 1–3 small joints (±large joints) corresponding to 2;
- 4–10 small joints (±large joints) corresponding to 3;
- >10 joints (≥1 small joint + any others) corresponding to 5;
- Negative RF and negative ACPA corresponding to 0;
- Low-positive RF and/or ACPA ≤3× upper limit of normal for local laboratory assay corresponding to 2;
- High-positive RF and/or ACPA >3× upper limit of normal corresponding to 3;
- Abnormal erythrocyte sedimentation rate (ESR) and/or abnormal C-reactive protein (CRP) corresponding to 1;
- Normal CRP and normal ESR corresponding to 0;
- Patient reported pain, swelling and tenderness ≥6 weeks corresponding to 1 [97].

Patients with a score of ≥6 are classifiable as having RA. To be eligible for a new series of tests, two mandatory conditions must be met. The first one is the need of evidence of synovitis, with a swelling in at least one joint as evaluated by a specialist, not including the typical joints involved in osteoarthritis: the first metatarsophalangeal joint, the first carpometacarpal joint and distal interphalangeal joint. The second condition for applying the criteria is that the patient does not have another diagnosis for synovitis. Moreover, the large joint category includes ankles, hips, elbows, shoulders, and knees, while the small joints category consists of proximal interphalangeal joints, wrists and second through fifth metatarsophalangeal joints [98].

Algorithms have been reported for the diagnosis of early RA, with different development, depending on the features of each patient [96]. As a general disease pattern, RA presents an insidious onset with gradual progression, being associated with joint pain, tenderness, swelling and symmetrical joint damage [98]. RA is predominantly observed in the elderly group and, if left untreated, it can lead to loss of function, disability, and an increased burden of disease [15,99].

Using differential diagnosis to confirm RA is a challenge and represents the optimal medical approach. In order to make a differentiation of RA from other similar diseases, certain features must be evaluated. A biopsy is required sometimes to differentiate diseases with similar conditions. The distribution of synovitis is different in RA (symmetric, great, and small joints including wrist and elbow) than in ankylosing spondylitis (limited to small joints) and psoriatic arthropathy (asymmetric, including toes). Inflammation is more intense in RA than in osteoarthritis [95]. The presence of RF is associated predominantly with RA, but also with Sjögren’s syndrome and SLE [100]. Antinuclear antibodies are more common in SLE than in RA. The most intense erosive changes on X-rays are found in RA. Cutaneous signs suggest SLE, PsA [101,102] or systemic sclerosis. PM usually primarily affects the shoulders and hips. In SpA, the most common inflammatory processes affect the eye and the back [95]. Patients who cannot be classified according to ACR-EULAR criteria due to a duration of symptoms of less than 6 weeks may be suspected of having a viral infection (*Parvovirus*, *Enterovirus*) or Lyme arthritis [103]. When more than four joints are affected by arthritis, the disease is called polyarthritis and it is difficult to differentiate it from osteoarthritis and fibromyalgia, when pain is the only symptom. Furthermore, blood and urine tests may help in establishing an accurate diagnosis. Other differences concerning extra-articular manifestations have also been reported [95].

#### 4.1.1. Diagnostic, Prognostic, and Predictive Biomarkers in RA

The identification and optimization of biomarker panels represents a promising medical tool, due to their diagnostic, prognostic, predictive and therapeutic role. The ACR 1987 criteria included only RF as a biomarker. The last established classification includes four biomarkers (RF, ACPA, ESR, CRP), all with certain limitations [104]. More recent studies have identified other diagnostic proteins with roles in early diagnosis of RA: antibodies against mutated citrullinated vimentin (anti-MCV), antibodies against carbamylated proteins (anti-CarP) and 14-3-3 eta protein. A systematic review of studies in which biomarkers were tested for their potential diagnostic role has reported no difference between cyclic ACPA and anti-MCV. Thereby, it can represent a diagnostic tool when RA and ACPA are negative [105]. Moreover, the diagnostic accuracy of 14-3-3 eta protein has been evaluated in experimental studies and it has been reported that for all RA patients with a negative RF and ACPA, 14-3-3 eta protein was positive [106,107]. Anti-CarP was detected in RA patient serum and several studies have reported that the presence of anti-CarP is associated with pre-symptomatic phases and may also be used as a prognostic tool [108,109,110].

More recent studies have demonstrated that gene profiles can represent important diagnostic tools. By comparing FLS from healthy individuals to FLS-RA it has been demonstrated that statistically significant differences can occur in heat-shock protein family A members, matrix metalloproteinase 1 (MMP1), matrix metalloproteinase 13 (MMP13) and tumor necrosis factor ligand superfamily member 10 (TNFSF10) genes [111,112,113,114]. Furthermore, the technical development of proteomics enables the identification of protein panels, with an important role in early diagnosis. An experimental study used label-free quantitative proteomics to characterize proteins with diagnostic potential, especially for seronegative RA patients. It has been demonstrated that serum amyloid A-4 protein (SAA4), retinol-binding protein-4 (RBP4), angiotensinogen (AGT) and vitamin D-binding protein (VDBP) are accurate enough to be used as diagnostic tools [115]. Glycoprotein YKL-40 can be used as a diagnostic biomarker because of its promising results [116]. A few biomarkers (anti-MCV, RF, 14-3-3 eta protein, ACPA) can also be used as prognostic tools because they are associated with severe phases of RA [117,118]. Further research is needed in order to identify new potential prognostic biomarkers.

Predictive biomarkers are essential in the therapeutic management of RA because they are used to establish an effective treatment that all patients will respond to. It has been reported by several studies that anti-CCP, anti-MCV, 14-3-3 eta, cartilage oligomeric matrix protein (COMP), survivin and calprotectin are correlated with a good predictability of treatment response [119,120,121,122].

#### 4.1.2. Imaging Diagnosis of RA

An accurate diagnosis involves the association between the detection and quantification of biomarkers with imaging tools. The ACR-EULAR 2010 classification includes ultrasonography, computed tomography (CT) and magnetic resonance imaging (MRI) as imaging tools for establishing an early diagnosis, due to their much higher accuracy than in the case of conventional radiographs [97]. X-ray examinations of joints cannot reveal the early presence of degradations and erosions [123]. Even though X-ray is still used as a diagnosis technique of late changes in the joints because of its availability, low cost, and more medical records, it has limitations due to the radiations that are used, low sensitivity in detecting early erosion processes and because 3D anatomical structures are shown only in 2D [124]. Moreover, a few radiographic hallmarks of RA have been identified, including symmetrical abnormalities, periarticular osteopenia, narrowing of the joint spaces and marginal degradation, swelling of the soft tissue and synovial cysts and nodules [125,126].

Ultrasonography is a diagnosis technique that characterizes the interaction between tissues and sound waves to produce an image of the tissue. It can detect small bone and cartilage erosions and explore the structures in great detail. Doppler ultrasound may differentiate active from inactive inflammatory tissues [127]. The inherent advantage of ultrasonography over X-ray has been demonstrated in a case-control study, where sonography detected more erosions, especially in early RA [128].

CT is a rarely used imaging technique which, due to its ionizing radiation, can damage the deoxyribonucleic acid (DNA) of human cells, and it has limited soft tissue contrast [126,129]. However, it can be successfully used in medical cases where 3D imaging is required. Clinical trials conducted over time have demonstrated similarities between CT and MRI [130,131]. However, MRI is the most accurate imaging tool for the detection of early RA. Contrast enhanced MRI can generate a differential diagnosis between joint effusion and synovitis. Furthermore, it can detect early erosions and hypertrophies and it is the gold standard for bone marrow edema detection. A recent longitudinal study evaluated the role of MRI in predicting RA progression in patients with clinical symptoms, but showed no correlation between them, even though the detection accuracy was high [132].

The utility of various imaging tools in RA depends on the RA stage of progression. MRI is the most suitable imaging method for the detection of early changes in RA patients, except for detecting joint space widening, where CT is more appropriate. For late changes that occur in the joints, all the imaging tools mentioned above can be used with good results. Future challenges and optimization strategies in medical imaging include thermography, near infrared imaging (NIR), positron emission tomography (PET) and single-photon emission computerized tomography (SPECT) [126].

### 4.2. Extra-Articular Disease Manifestation in RA

RA is an autoimmune disease that primarily affects the small joints and then the large ones, but as a systemic disorder, extra-articular structures can be involved. Affected joints may be in the upper extremity (hand, wrist, elbow, shoulder), lower extremity (foot and ankle, forefoot, midfoot, hindfoot, knees, hips) and spine and axial joints (C-spine, atlantoaxial subluxation, basilar invagination, sub axial subluxation, thoracic spine, sternoclavicular spine, manubriosternal joints, lumbar spine, temporomandibular joint, sacral spine, cricoarytenoid joint, ossicles of the ear). Extra-articular manifestations (EAMs) in RA are serious conditions correlated with high morbidity and mortality rates. EAMs may result from the release of proinflammatory cytokines in the bloodstream [133].

Various tissues and organ systems can be affected. The most severe manifestations include vasculitis, Felty’s syndrome, pericarditis and pleuritis and it has been reported in a retrospective study conducted in a cohort of 424 cases, that 39.85% of patients developed severe EAMs [134]. Moreover, a multicenter research trial evaluated the frequency of EAMs in 587 RA patients and showed that 40% of patients developed extra-articular features [135].

Systemic vasculitis may result in skin manifestations, gastrointestinal complications, cardiac disease, and pulmonary manifestations. The most common skin manifestations are rheumatoid nodules located in different areas, which can occur mainly in seropositive patients with erosive disease. Other skin manifestations include periungual inflammation, ulcerations and digital gangrene [2]. Ocular manifestations are not as common as those on the skin and include kerato-conjunctivitis sicca as the most frequent manifestation of this subset, episcleritis, scleritis and keratitis. The swelling of the salivary gland and xerostomia are oral manifestations that can occur. However, ocular and oral manifestations can also be found in Sjögren’s syndrome [133].

Pulmonary complications are frequent, but asymptomatic, including pleural effusions, pulmonary fibrosis, interstitial lung disease and arteritis. Smokers are at greater risk of developing life-threatening complications of RA [2].

RA patients can be associated with an increased risk of cardiovascular mortality because numerous cardiac structures are involved in the pathological processes, which may lead to atherosclerosis, arterial stiffness, coronary arteritis, congestive heart failure, valvular disease and fibrinous pericarditis [136]. It could potentially contain prognostic markers of diseases like hypertension and dyslipidemia [137].

It has been reported in a meta-analysis of 14 controlled observational studies involving 41,490 patients, that the risk of CVD increased by over 48% in RA patients compared to the general population [138]. Moreover, a case-control study assessed potential cardiac abnormalities in 47 RA patients without manifested cardiovascular symptoms using Doppler echocardiography technique and showed a high incidence of pulmonary hypertension and left ventricular diastolic dysfunction [139].

Renal manifestations are rare, including glomerulonephritis and interstitial renal disease, which are correlated with the presence of vasculitis, while neurological complications may result in peripheral neuropathy and cervical myelopathy [140].

The most common hematologic abnormality in RA patients is anemia, due to hepcidin stimulation that inhibits iron transport. Moreover, it has been reported that hepcidin may be a valuable prognostic biomarker in RA [141]. Other EAMs include malignancies, neutropenia, eosinophilia, and thrombocytopenia [2].

Felty’s syndrome is a severe EAM, which can occur mainly in seropositive patients with a low white blood cell count and an enlargement of the spleen. Thus, these patients are more susceptible to opportunistic infections [142].

Since both RA and ageing are linked to emerging comorbidities, such as cardiovascular disease (CVD), infections, interstitial lung disease and cancer, these elements will have a significant impact on RA global management. Moreover, knowing the current status of the management of RA-associated comorbidities and the formation of a multidisciplinary team of medical specialists, are essential parts in an attempt to lower morbidity and mortality rates [143].

The RBSMR study managed to define the profile of comorbidities by following 225 RA patients. The prevalence of CVD was 23.1% and of pulmonary disease was 5.77% [144].

Comorbidities and associated risk factors should be screened for and evaluated on a regular basis, as well as the management of these conditions. Lifestyle advice about regular physical activity, balanced diets, quitting smoking and vaccine updates should be parts of the management program.

Controlling the inflammatory process with DMARDs, especially targeted therapy, is linked to a lower risk of CVD [145,146]. A recent cross-sectional real-life study reported that the use of less glucocorticoids and an increasing use of bDMARDs in patients with cardiovascular comorbidities suggested that rheumatologists have become aware of the potential influence that RA drugs may have on comorbidities [147].

Current cardiovascular risk management involves the evaluation of conventional cardiovascular risk factors (diabetes, obesity, dyslipidemia, hypertension) using HeartScore^®^ and the control of inflammatory states. According to EULAR recommendations, the rheumatologist is in the most suitable position to organize risk factor assessment and care in RA patients [148]. The results of the cardiovascular risk assessment guide the LDL-cholesterol level, the frequency of testing, and show whether a cardiologist’s opinion is necessary. Moreover, according to EULAR, the presence of carotid artery plaque implies a high level of cardiovascular risk. About 60% of RA patients have carotid artery plaque [149]. However, scientific evidence indicates that statins reduce RA-related cardiovascular risk by reducing cholesterol, but also provide angioprotective, anti-inflammatory and antioxidative effects. Because of this, as well as their safety profile, statins can be a good option for comprehensive control of RA vascular comorbidities [150].

The measurement of cardiovascular risk is only one aspect of comorbidity screening and management. Infections, lung disease and malignancies are among risks associated with RA, which can be exacerbated by RA or its treatments [151].

Immunization is a very important aspect to consider because the risk of infection is given both by the pathology per se, but especially by immunosuppressive agents. In RA patients, the influenza vaccine should be given annually, diphtheria-polio-tetanus every 10 years and the pneumococcal vaccine every 5 years, in accordance with the vaccines recommended for the general population [152].

In RA patients, lung disease is one of the most common causes of extra-articular morbidity and mortality. The six-minute walk test and the five-point Medical Research Council breathlessness scale should be used in clinical evaluations to quantify exercise tolerance [153]. Due to the limited evidence, there are no international guidelines for the treatment of interstitial lung disease, as a comorbidity of RA. Therefore, the management of RA patients with moderate to severe lung disease should include a collaboration with a respiratory physician, especially given that evidence shows serious respiratory adverse events when using drugs for the treatment of RA [154].

Screening tests for malignancies applied to the general population are also useful for RA patients. Mammograms are the most suitable diagnostic tools for detecting breast cancer, the Pap and the HPV test for detecting cervical cancer and low-dose computed tomography for lung cancer screening [155].

The existing medical evidence showed that the management of comorbidities is not efficient enough and further research is needed. Incorporating comorbidities into the daily management of RA would lead to a more complete standard of care and screening tests should be included in diagnostic procedures for RA patients. The Canadian Dermatology-Rheumatology Comorbidity Initiative provided 19 evidence-based recommendations for managing comorbidities in RA patients, emphasizing the importance of differentiating comorbidities due to RA per se from those caused by therapeutic agents [156].

A few cohort studies have reported decreases in the incidence and prevalence of EAMs of RA in the last decades [134,157,158]. The management of EAMs in RA has improved as technological and medical advances have emerged providing a better understanding of the mechanism underlying the effects.

## 5. Therapeutic Approaches in RA

Different treatment strategies have been used over time in order to improve patients’ quality of life, to reduce the risk of EAMs and to determine the safety and efficacy profile of new active molecules. The principle established by the ACR is “Treat to target”, which refers to the choice of a good treatment to achieve remission, or a reduced disease activity as an alternative. Therapeutic intervention must be aggressive and rapid because already existing erosions are not reversible [97]. The general approach to treatment starts with a highly accurate diagnosis and includes prevention strategies, nonpharmacological and pharmacological therapies, in order have a quick result. The 2021 ACR guideline for the treatment of RA updated the pharmacological management of RA, providing seven strong recommendations and 37 conditional ones [159].

### 5.1. Nonpharmacological Interventions for RA

The characterization of risk factors provides tools for preventing RA. Focusing on prevention may be an important part of the general management of RA. Four levels of prevention (primary, secondary, tertiary, clinical) have been shown. Primary prevention is focused on not allowing pathological processes to begin, the secondary one manages the risk factors to detect and reduce them, and tertiary prevention deals with damage-limiting mechanisms. Clinical prevention includes reducing complications and stopping relapses [160]. Screening strategies of people at risk of developing RA may result in lower incidence and prevalence rates. Blood relatives, twins of RA patients and seropositive individuals should be closely monitored because they are in the risk category [161].

The goals of nonpharmacological approaches are to decrease anxiety and depression, to reduce pain and to increase mobility. Polyunsaturated fatty acids (PUFAs) have gained wider attention because of their links to a variety of brain disorders, including anxiety and depression. These PUFAs include docosahexaenoic acid (DHA) and eicosapentaenoic acid (EPA) in the series of omega-3 fatty acids [162]. To determine the efficacy of PUFAs, particularly DHA and EPA, in the treatment of depression, a meta-analysis of 26 double-blind randomized placebo-controlled trials was conducted. The results showed that omega-3 PUFAs improved depression significantly. The ratio of EPA to DHA with the most effective antidepressant properties was 2:1 or 3:1 [163]. In addition, two meta-analyses estimated that the most effective formulations are those containing ≥60% EPA [164,165]. Another meta-analysis of 19 clinical trials assessed the potential of PUFAs to relieve anxiety symptoms. Existing medical evidence suggested that PUFAs may influence several neural processes that underlie anxiety. Even though diagnoses were diverse, the major conclusion was that omega-3 PUFAs were linked to a significant reduction in anxiety symptoms when compared to controls [166]. Moreover, a recent prospective study including 36 patients with JAKi treatment discovered an inverse association between patients’ pain scores and DHA serum levels and showed that pain relief can be promoted by supplementation with PUFAs. Due to the small number of patients included in the study, large prospective studies need to be done in order to confirm the hypothesis [167].

In RA patients, anxiety, depression, and pain are associated with disease activity and a poor functional status. According to the medical evidence existing so far, PUFAs can become useful tools in controlling the symptoms, but additional studies are needed.

Rest, occupational therapy, physical exercise, and surgery can also be useful. Most studies that have evaluated the role of physical activity and psychological interventions for RA-related fatigue patients, have demonstrated their effectiveness and associated with rest, they may relieve stress on inflamed tissues and slow down the progression of the disease [168,169]. A systematic review of 42 articles about the advantages of occupational therapy for RA patients showed an increase in joint function [170].

Joint surgery is used only in severe stages of RA. However, the rates of surgery in RA have low values in patients under 60 years. Surgical approaches provide pain relief and restore the function of joints. Due to recent advances in the surgical field, numerous procedures are available: tens-synovectomy, radio synovectomy, arthroscopy, osteotomy, joint fusion, metatarsal head excision arthroplasties or total joint replacement [171]. Scientific evidence suggests that massage, positioning, hot and cold therapy, acupuncture, transcutaneous electrical nerve stimulation and progressive muscle relaxation are complementary therapies that might be useful in nonpharmacological pain management [172]. Nonpharmacological approaches should be associated with pharmacological treatments in order to maximize therapeutic success.

### 5.2. Pharmacological Therapies in RA

Continuous improvement in the procedures and techniques in drug design strategies has led to considerable progress in pharmacological approaches towards finding a cure for RA. The new therapeutic options have managed to reduce the symptoms, slow the progression and prevent complications. Current treatment options in accordance with ACR and EULAR recommendations manage RA from two perspectives: symptomatic treatment (NSAIDs and GCs) and disease modifying management (DMARDs) [159,173].

The symptomatic management of RA consists primarily of NSAIDs and GCs, but weak opioid analgesics may also be considered for short-term management of pain after an accurate assessment of the benefit–risk balance [14,174].

NSAIDs (naproxen, ibuprofen, coxibs) are used in the acute phase response to reduce pain by decreasing inflammation. NSAIDs exert their pharmacological effect by inhibiting cyclooxygenase (COX), especially COX-2 which is increased during inflammation. However, the risk of harm should be considered because the inhibition of prostaglandins can lead to serious side effects, such as bleeding, gastrointestinal ulceration, renal failure, heart failure, rashes, dizziness, confusion, seizures, etc. Some of the side effects can be avoided by using COX-2-selective NSAIDs (celecoxib, rofecoxib, valdecoxib) [14,175]. The effectiveness of NSAIDs in RA has been demonstrated in placebo-controlled trials in which patients without GC treatment were included [176].

GCs (prednisone, hydrocortisone, prednisolone, dexamethasone) have greater potency and efficacy than NSAIDs, due to the complex mechanisms of their anti-inflammatory and immunosuppressive effects, but the safety profile of NSAIDs is slightly better [177]. Long-term side effects of GCs include weight gain, water retention, muscle weakness, diabetes, bone thinning, etc. Thus, they have a short-term use and can be administered orally, intravenously, intramuscularly, and intra-articularly [178]. GCs have two major roles in the treatment of RA, as bridging therapy for DMARDs until their effects start and as adjunctive therapy for active RA that persists despite using DMARDs. It is critical not to abruptly discontinue corticosteroid therapy due to negative feedback in the regulation of hypothalamic–pituitary–adrenal (HPA) axis pulsatility [177].

DMARDs are pharmacological agents that are used to promote remission by suppressing autoimmune activity and by delaying or preventing joint degeneration. The treatment should be initiated as soon as possible because early implementation leads to better results, especially given that DMARDs are slow acting drugs with a delayed onset of between 6 weeks and 6 months. DMARDs have been classified as conventional synthetic DMARDs (csDMARDs), biologic DMARDs (bDMARDs) and targeted synthetic DMARDs (tsDMARDs) [179]. csDMARDs are typically used as a first-line therapy for newly diagnosed RA patients. bDMARDs or tsDMARDs are recommended if first-line therapy is not tolerated or is ineffective. tsDMARDs, including the class of Janus kinase inhibitors (JAKi), have the advantage of being orally administered [180].

csDMARDs are a heterogeneous class of drugs including methotrexate (MTX), leflunomide (LEF), hydroxychloroquine (HCQ) and sulfasalazine (SSZ), which are more frequently used than other agents with a lower efficacy and safety profile, such as gold salts, azathioprine, d-penicillamine, cyclosporine, minocycline, and cyclophosphamide. Their mechanisms of action lead to a non-targeted suppression of the overactive immune system [12,177].

The 2021 ACR guideline for the treatment of RA claims MTX as a first-line treatment for RA, both as a monotherapy and associated with other molecules as well, due to its efficacy and safety profile, flexible administration, and low cost. Moreover, the guideline strongly recommends the use of MTX monotherapy over hydroxychloroquine, sulfasalazine, bDMARDs, and tsDMARDs for RA DMARDs-naive patients with moderate-to-high inflammatory activity. Furthermore, its conditional recommendations include the use of MTX over LEF for RA patients untreated with DMARDs, and the use of MTX monotherapy over dual or triple csDMARDs therapy or over MTX associated with bDMARDs or tsDMARDs. The inhibition of purine biosynthesis and cytokines production, as well as the activation of adenosine receptors lead to the anti-inflammatory properties of MTX. Oral administration of MTX is conditionally recommended over other administration routes for DMARDs-naive patients [159]. A recent systematic review of 73 clinical trials assessing the efficacy and safety profile of MTX showed it had the safest profile of any csDMARDs used for RA and great efficacy rates [181,182]. Toxic effects identified over time are rare and are mainly gastrointestinal, hepatic, hematologic and pulmonary, consisting of diarrhea, nausea, liver damage with a lower incidence of cirrhosis, thrombocytopenia, leukopenia, pulmonary fibrosis, and pneumonitis [183].

A meta-analysis performed in order to compare the efficacy and safety profiles of LEF and MTX demonstrated the similarity of their efficacy profiles and a slightly lower safety profile for LEF, due to a higher increase in liver enzymes [182]. Thus, LEF can be used as an alternative initiating treatment option for patients with poor tolerance to MTX [184].

HCQ is a drug used for malaria, but due to its immunomodulatory effects with a decreased secretion of cytokines, can be an alternative option in the treatment of RA. A multicentric, randomized, double-blind, placebo controlled clinical trial assessed the efficacy and safety of HCQ in RA and demonstrated that the drug was effective and well-tolerated in the patients included in the study [185]. The principal benefit of HCQ is that it has no myelosuppressive, renal or hepatic side effects. However, at higher dosages, the eye can be affected, and pre-retinopathy can develop [177,186].

SSZ is a two-metabolite prodrug with anti-inflammatory and immunosuppression effects. Its similar efficacy to LEF has been demonstrated in a multicentric, randomized, double-blind, placebo controlled clinical trial, but the use of SSZ is limited by its side effects, such as rash, serum sickness-like reactions, urticaria, nausea, and diarrhea. Serial monitoring of certain laboratory tests and managing the changes that can occur may reduce the side effects [12].

**Figure 4:**
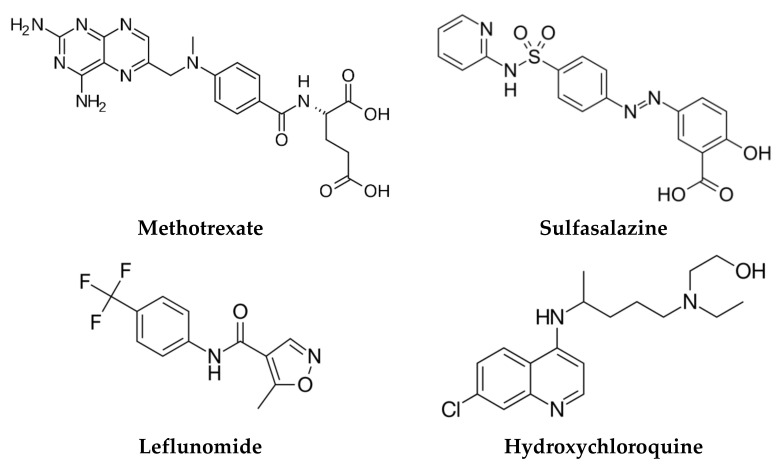
Molecular structure of the most widely used csDMARDs.

For DMARD-naive patients with low disease activity, the 2021 ACR guideline for the treatment of RA suggests that HCQ is conditionally indicated over other csDMARDs, SSZ is conditionally suggested over MTX, and MTX is conditionally recommended over LEF. The chemical structures of the most prescribed csDMARDs are depicted in Figure 4 [187].

Further treatment options including bDMARDs, tsDMARDs, biosimilars or combination therapy are available when csDMARDs are ineffective or poorly tolerated. bDMARDs are a newer option for the treatment of RA and provide a targeted therapy on the structures of the immune system [12]. bDMARDs are genetically engineered protein molecules divided into several classes, depending on the mechanism of action, as follows:

- TNF-α inhibitors (etanercept, infliximab, golimumab, adalimumab, certolizumab pegol);
- B-cell depleters (rituximab, ofatumumab);
- B-cell receptor inhibitors (belimumab, atacicept, tabalumab);
- Antagonists of CD28 on T-cells (abatacept, belatacept);
- IL-1 inhibitors (anakinra, canakinumab, rilonacept);
- IL-6 inhibitors (tocilizumab, sarilumab, sirukumab, olokizumab, clazakizumab);
- IL 12/23 inhibitor (ustekinumab);
- IL-17 inhibitors (ixekizumab, secukinumab, brodalumab);
- Granulocyte-macrophage colony-stimulating factor inhibitor (mavrilimumab, otilimab);
- RANKL inhibitor (denosumab) [12,159,188,189].

Since their discovery, the use of bDMARDs has been on an upward trend. This statement is supported by a study the aim of which was to analyze prescription patterns for csDMARDs and bDMARDs between 2004 and 2011 by using yearly cross-sectional investigations [189,190]. The use of csDMARDs is still much higher than bDMARDs. Over the 7 years of the study, there was a permanent increase in the use of csDMARDs, from 6.53% in 2004 to 8.93% in 2011. The study also showed a significant increase in the annual prevalence of bDMARDs use from 2004 (0.35%) to 2011 (1.54%). The most prescribed bDMARDs were adalimumab (between 0.07–0.35%), etanercept (between 0.16–0.46%) and rituximab (between 0.03–0.21%). Moreover, the prevalence of prescriptions has increased over the years, except for anakinra for which a constant value has remained (0.01%) [189].

A randomized, double-blind, placebo controlled, phase III clinical trial evaluated the efficacy and safety profile of adalimumab as a monotherapy in patients with RA who had failed to respond to csDMARDs [191]. The results showed both statistically significant improvement in the disease activity and a good safety profile. However, due to the suppression of the immune system, bDMARDs influence the susceptibility to infections and this aspect should be carefully monitored. A meta-analysis of nine clinical trials of adalimumab in the treatment of RA demonstrated its association with a greater risk of serious infection, which increased with dose [192]. One of the shortcomings of bDMARDs, especially TNF-α inhibitors, is the risk of developing tuberculosis (TB). The use of TNF-α inhibitors was associated with an 18-fold increased TB incidence in a population-based cohort investigation of RA patients from a high-incidence area. When compared to etanercept, adalimumab was linked to a higher and earlier diagnosis of TB [193]. Adalimumab-atto, adalimumab-adbm, adalimumab-adaz, adalimumab-bwwd, adalimumab-afzb and adalimumab-fkjp are biosimilars approved by the FDA for the treatment of RA [194].

Etanercept was the first anticytokine medication approved by the FDA for RA treatment [195]. It is the only TNF-α inhibitor that is not an antibody, but a dimeric fusion protein. A long-term evaluation of the safety and efficacy profile of etanercept in 549 RA patients was made through an open-label trial, which showed that after 36 months of treatment, etanercept demonstrated long-term efficacy, as well as a favorable safety profile [196]. It is administered twice weekly via subcutaneous injection and has a toxicity profile like infliximab. It has also demonstrated a beneficial role in reducing radiographic progression in RA patients. According to the medical literature, the number of patients who achieved clinical remission with etanercept varied between 50% and 75%. Etanercept-szzs and etanercept-ykro are biosimilars approved by the FDA for the treatment of RA. Even though a meta-analysis of all Cochrane reviews on bDMARDs for RA estimated that adalimumab, etanercept and infliximab had similar efficacy profiles [197], etanercept had the best drug survival of all TNFi, according to the SSATG and DANBIO registries [198].

As bDMARDs become more widely used and for longer periods of time, studies of their long-term safety and efficacy are becoming increasingly important. An example of such a study was conducted in order to assess the safety and efficacy profile of etanercept beyond 10 years of therapy in 1272 North American RA patients treated with 25 mg of etanercept twice a week for 10 years. The study reported 5 opportunistic infections, 29 cases of sepsis, 14 lymphomas and 61 deaths, but the occurrence of serious adverse events was higher in longstanding RA patients that in early RA patients. However, it was demonstrated that etanercept provided a good risk/benefit ratio due to its efficacy and safety profile and can be a long-term therapeutic option [199].

Infliximab is a chimeric monoclonal antibody with a human antibody backbone that binds to all forms of TNF-α, neutralizing its biological function. It is administered by intravenous infusion. A decrease in adhesion molecules, IL-1, IL-6 and IL-8 was found after therapy with infliximab in RA patients [12].

Medical evidence indicates that patients treated with infliximab have a quick response and it has a good preventive effect on joint degeneration. A cohort study assessed 24 cases of RA patients with medium and high disease activity, despite the use of bDMARDs like adalimumab, golimumab, tocilizumab, etanercept or abatacept. The medical intervention was the switch to infliximab and the results showed a good efficacy profile due to the 37.5% of patients who achieved a low disease activity and 70.8% of patients who achieved a moderate or good EULAR response [200]. The safety profile was also good because only one serious adverse event was identified (infection with hospitalization). Infliximab-dyyb, infliximab-abda, infliximab-qbtx and infliximab-axxq are biosimilars approved by the FDA for the treatment of RA. Recent studies demonstrated that there are no statistically significant differences in terms of efficacy and safety between bio-original and infliximab biosimilars [201].

Golimumab is a human monoclonal antibody administered once a month by subcutaneous injection. Even though it has a similar safety and efficacy profile to other TNFi, golimumab is less effective than other TNFi in individuals who have failed multiple biological treatments. However, due to its high mass it can be a good therapeutic option during lactation. The biosimilar products of golimumab are still in a preclinical phase [202].

Certolizumab is a human monoclonal antibody administered every 2 weeks by subcutaneous injection. It is a biological molecule that can be safely used during pregnancy due to its lack of placental transfer and it has been approved for the treatment of RA in pregnant women [203]. The biosimilar products of certolizumab are still in a preclinical phase [202].

Abatacept is a fusion protein that inhibits T cell activation by blocking the interaction with CD28. It is administered by intravenous infusion and should be administered 2 and 4 weeks after the first infusion, then every 4 weeks. Numerous phase 3 trials have examined the safety and efficacy profiles of abatacept. A double-blind trial including RA patients with a poor response to MTX therapy assessed the safety and efficacy of abatacept or infliximab versus placebo. The results of the study estimated a similar efficacy profile of abatacept and infliximab, but a better safety profile for abatacept, with fewer adverse events [204]. However, an observational post-marketing study analyzed individual case safety reports provided by VigiBase, to compare the incidence of cancer reported in RA patients receiving abatacept compared to those receiving other bDMARDs. Abatacept was only substantially related with an elevated risk of reporting melanoma in RA patients when compared to other bDMARDs [205].

Tocilizumab is a monoclonal antibody with an IL-6 inhibition mechanism. It is available on the pharmaceutical market as an infusion and can be administered subcutaneously and intravenously. Evidence from 14 phase 3 clinical trials suggested that the immunogenicity risk of tocilizumab is low, regardless of the route of administration [206]. According to the ADACTA study, tocilizumab therapy was found to be more effective than adalimumab monotherapy in terms of reducing signs and symptoms in RA patients with an inadequate response to MTX therapy [207]. The most common side effects reported in clinical trials are upper respiratory tract infections, nasopharyngitis, cellulitis, and high blood pressure [202].

Rituximab is a well-tolerated molecule, not associated with an increased risk of infection. To evaluate infection rates between rituximab and placebo, a fixed-effect meta-analysis was conducted. The study’s findings showed that the risk of serious infection when rituximab is administered is low, even at higher doses [208]. Furthermore, rituximab is a monoclonal antibody with good efficacy in RA as demonstrated by a prospective, noninterventional study and can be an alternative for patients with an inadequate response to treatment with MTX or TNF-α inhibitors [209].

A network meta-analysis of the most suitable Cochrane reviews on bDMARDs for RA compared the efficacy and safety profile of six bDMARDs (abatacept, adalimumab, anakinra, infliximab, rituximab, etanercept). The results showed that adalimumab and etanercept were more effective than anakinra, and that adalimumab, infliximab and anakinra are less safe than etanercept [197].

bDMARDs are scientific breakthroughs that have revolutionized the treatment of RA. Numerous benefits have been reported in RA patients who had a poor response to csDMARDs. The focus of therapeutic management is on a rapid and aggressive intervention with the most effective drugs. The high cost of biologics is one the major factors limiting patient access to bDMARDs. However, patient profiles have changed over time and now include shorter disease progression times and lower disease activity. A trend of prescribing bDMARDs as the first line of treatment in RA patients with comorbidities has been observed [210].

The newest therapeutic approach to RA approved by the FDA and EMA involves the use of JAKi. These molecules are divided into two groups based on their selectivity, the first one consisting of inhibitors with low selectivity, inhibiting the signaling of a broad range of cytokines, and the second generation that can selectively inhibit signaling processes. JAKs are cytoplasmic proteins that connect cytokine signaling from membrane receptors to transcription factors known as signal transducers and activators of transcription (STAT), so that an optimal control of the inflammatory response can be achieved, and they can also become a valuable tool for the management of autoimmune diseases [211]. Moreover, there are four members in the JAKs family (JAK1, JAK2, JAK 3 and tyrosine kinase 2, TYK2) and seven types of STATs (STAT1, STAT2, STAT3, STAT4, STAT5A, STAT5B, STAT6), that can be targets for JAKi [212]. In addition to the good efficacy and safety profiles, other important advantages of JAKi are their oral route of administration and that their production costs are lower than those of bDMARDs [213,214,215].

The 2021 ACR guideline for the treatment of RA updated the recommendations on using JAKi when csDMARDs are ineffective. Furthermore, patients’ compliance to JAKi monotherapy may be higher than with multiple therapies with csDMARDs, in this case with the safety profile being lower [159,216]. Due to the suppression of the immune system, the risk of infections, especially pulmonary ones, can be high and therefore vaccination before initiating treatment is recommended [217,218]. There has also been a reported dose-dependent alteration of lipid metabolism, but without a correlation with an increased risk of cardiac disease [212]. Table 2 provides an overview of JAKi, emphasizing their novelty in RA management and presenting their molecular targets and safety profiles [212,219].

Clinical trials have provided complex information on the safety and efficacy profile of JAKi and an increase in their future use is expected as the pathophysiological processes of RA and the pharmacological properties of therapeutic agents are fully elucidated. In this context, results of post-marketing surveillance (PMS) are becoming an important tool for ensuring that therapeutic approaches remain safe and effective but can also reveal areas where improvements can be made. To assess the safety of tofacitinib, baricitinib and upadacitinib in a post-marketing setting, researchers analyzed PMS reports and then provided valuable information.

A meta-analysis systematically assessed tofacitinib PMS data obtained in the Pfizer safety database from November 2012 (approval date) to November 2015. Reporting rates (RR) were computed by dividing the number of serious adverse events by the predicted 100 patient-years of exposure. Moreover, patient exposure was determined by using estimated worldwide sales and a twice-daily regimen of tofacitinib 5 mg. Worldwide post-marketing exposure to tofacitinib since approval was estimated to be 34,223 patient-years, throughout the 3-year reporting period. In total, 9291 case reports were analyzed (82.9% non-serious) and 102 fatal cases were reported. The calculated RRs were 2.57 for infections, 0.45 for neoplasms and 0.43 for cardiac disorders. The most frequently reported adverse events out of a total of 25,417 reports were drug ineffectiveness (13.2%), headache (9.0%) and pain (6.4%). The types of events analyzed from the PMS data for tofacitinib in RA were correlated with the established tofacitinib safety profile, with no novel safety hazards identified [225].

Another recent meta-analysis of observational studies evaluated the risk of malignancy in RA patients exposed to non-TNFi or tofacitinib therapy, in comparison with csDMARDs or TNFi. A total of 10 studies out of 2819 identified articles involving 40,587 patients exposed to non-TNFi and 2221 patients exposed to tofacitinib were included. Abatacept exposure was associated with a minor risk of developing cancer, but tofacitinib, rituximab and tocilizumab were not associated with an elevated cancer risk [226]. However, a recent phase 4 study conducted between 2014 and 2020 assessed the safety of tofacitinib versus adalimumab and etanercept, in terms of major adverse cardiovascular events (MACE) and malignancies, excluding non-melanoma skin cancers. The goal of this study was to show the non-inferiority of tofacitinib compared to TNFi related to these pathologies. Results showed that the non-inferiority criteria that had been set were not met [227]. Furthermore, in PMS studies, JAKi, particularly tofacitinib, have been linked to an elevated risk for venous thromboembolisms [228]. However, this increased risk was only observed when tofacitinib was given at a dose of 10 mg twice a day, which is higher than the level recommended for RA in most countries [229].

At the moment, baricitinib is used less than tofacitinib. An all-case PMS study of baricitinib analyzed its safety and efficacy profile in Japanese patients with RA from September 2017 to June 2020. The data collected demonstrated no additional safety issues, indicating that baricitinib should be used in accordance with guidelines [230].

Because JAKi represent new therapeutic options, most phase 4 studies are ongoing. Two major phase 4 studies designed to compare the safety of baricitinib versus TNFi with respect to venous thromboembolic events are currently ongoing [231,232]. Moreover, there are also two ongoing studies on the use of upadacitinib. The first one started in October 2020 and assessed the change in disease symptoms in adult Canadian participants with moderate to severe RA (CLOSEUP). The estimated enrollment in multiple sites within Canada is about 390 participants and the estimated study completion date is September 2024 [233]. The second phase 4 study is more recent (January 2021) and larger (3000 participants). It is a PMS to evaluate the efficacy and safety profile of upadacitinib in Korean adult participants with RA [234]. Additionally, PMS are very necessary, especially for new therapies, and the data they bring will further guide JAKi therapy.

Due to the current epidemiological context, studies have been conducted to determine whether there are certain correlations between RA and coronavirus disease 2019 (COVID-19), starting from the evaluation of the cytokine storm and other data that showed an increase in the serum ACPA level after infection with SARS-CoV-2 [235,236]. Even though RA patients appeared to be more vulnerable due to their autoimmune disorder, the epidemiological parameters and the evolution of SARS-CoV-2 infection are not different from those reported in the general population, according to data from cross-sectional and cohort studies published so far. Furthermore, immunosuppressive agents do not appear to be linked to COVID-19 progression, so that the treatment of non-infected patients can carefully continue. However, further research is needed in order to investigate the influence of RA medications for patients infected with SARS-CoV-2, which is still controversial, so monitoring each therapeutic stage becomes essential [237].

## 6. New Perspectives and Future Directions in the Treatment of RA

**Figure 5:**
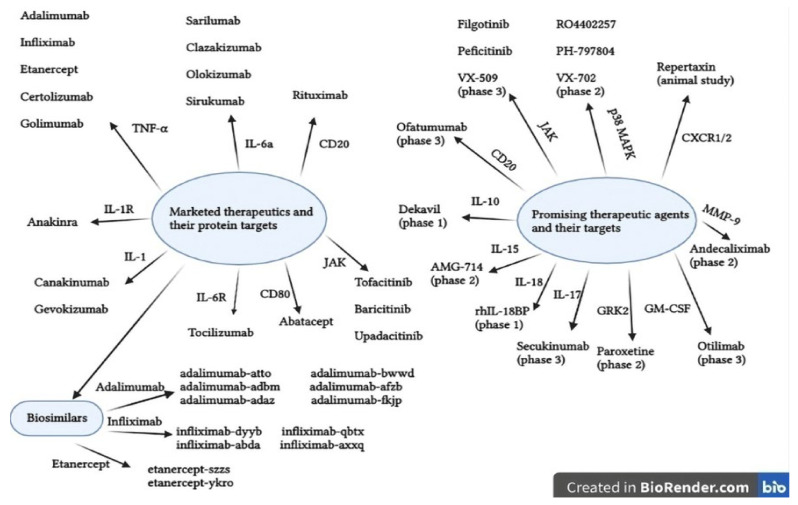
Status and future targeted therapies in RA. AMG, human monoclonal antibody; CD20, membrane-embedded surface molecule; CXCR, α-chemokine receptor; IL, interleukin; CD80, ligand for the protein CD28; JAK, Janus kinase; MAPK, mitogen-activated protein kinases; MMP, matrix, metallopeptidase; TNF-α, tumor necrosis factor alpha.

The management of RA has changed significantly over the last decades, resulting in improved quality of life and outcomes for RA patients. This has been made feasible by the successful discovery of several pathways involved in the pathogenesis of RA. However, both the mechanisms underlying the inflammatory processes and the pharmacological effects of therapeutic molecules are still incompletely elucidated, leading to some unmet needs in the management of RA. These include a deeper understanding of how different therapies have such comparable efficacies; elucidating why certain patients become less responsive over time; detecting pre-RA and establishing an early and aggressive treatment; and improving the efficacy and safety profiles of novel compounds, especially JAKis [31,238]. Several ways to improve RA treatment are now being tested in different experimental models. Numerous new therapeutic targets are being researched and potential therapeutic agents are in various stages of testing in order to obtain a complete remission of RA. The present and future of targeted therapies are summarized in Figure 5 [12,219].

Huang et al. (2021), updated and considered complex information about small molecular metabolite targets (prostaglandins, thromboxane A2, leukotriene B4 receptor, platelet-activating factor, cannabinoid receptors, inducible nitric oxide, etc.) and epigenetic targets (DNA methylation, RNA methylation, histone modification, etc.) and other protein targets (p38 mitogen-activated protein kinase, complex G protein-coupled receptor kinase 2, granulocyte-macrophage colony-stimulating factor), along with potential therapeutic agents [219].

Mesenchymal stem cells (MSCs) are also a promising therapeutic approach due to their ability to differentiate into new tissues like bone and cartilage and they have been reported to have immunosuppressive properties in vitro by suppressing T cell activation. Moreover, treatment with MSCs in both animal model studies and clinical trials in RA patients have shown a decrease in the proinflammatory response and an improvement of RA symptoms by reducing the blood levels of IL-1, IL-6, IL-8 and TNF-α [239].

Toll-like receptor 4 has a confirmed role in RA pathogenesis, promoting joint inflammation. Thus, therapeutic compounds targeting this receptor or its ligands, such as heat-shock protein crystalline or tenascin C can be optimized [239]. Therapeutic options for RA are increasingly diverse, and numerous ongoing studies can contribute to a significant improvement for RA patients by discovering new molecular targets, new therapeutic agents, and new methods to counteract side effects. A personalized approach based on genetic studies doubled by evidence-based medicine can transform the future of medicine and succeed in curing the incurable [240].

## 7. Conclusions

In this review, an overview was presented of the management of RA, centralizing updated information. In recent years, interest in controlling autoimmune disease has grown, with numerous studies testing various approaches to patients with RA. Even though it is still incurable, patients’ quality of life has improved considerably. Disease prevention strategies, screening programs of people at risk of developing RA and comprehensive information on the disease monograph provided to the population can significantly improve epidemiological parameters [12].

The first step to effective disease management is an early and correct diagnosis, especially as several signs and symptoms are also associated with other diseases. The proper use and interpretation of ACR-EULAR criteria, the identification and quantification of diagnostic biomarkers and the association of the obtained results with imaging techniques contribute to the establishment of an accurate diagnosis. According to existing data, the benefits of nonpharmacological interventions are greater the faster the diagnosis is established [239].

The ultimate goal of RA management is to start an aggressive drug treatment in order to obtain full remission or at least a significant reduction in symptoms and clinical signs. The results of the conducted studies facilitated the understanding of the pathophysiological mechanisms and developed new therapeutic approaches, which made RA become a manageable pathology. However, many RA patients continue to be unresponsive to current medications. There are still insufficient data to achieve complete control of the disease, highlighting the need for new drugs to be developed and a greater focus on personalized medicine [240].

## Figures and Tables

**Table 1:** Variation in the prevalence ratios of RA over time.

Country	Study Year	Prevalence Ratio (%) (95% Confidence Interval)	Variations	Ref.
Serbia	1991	0.18	0.17	[16]
	2013	0.35		[22]
Italy	1991	0.33	0.07	[19]
	2011	0.4		[23]
Japan	1996	1.7	−0.95	[20]
	2016	0.75		[24]
China	1997	0.28	0.14	[17]
	2013	0.42		[25]
Argentina	1998	1.97	−1.03	[21]
	2010	0.94		[26]
France	2001	0.31	0.03	[18]
	2013	0.34		[22]
Spain	2002	0.5	0.32	[27]
	2017	0.82		[28]
Turkey	2004	0.49	0.07	[29]
	2017	0.56		[30]

**Table 2:** A brief characterization of JAKi.

JAKi	Generation	Molecular Target	Current Status	Most Frequent Side Effects	Ref.
Tofacitinib	I	JAK 1; JAK 2; JAK 3; TYK 2	Approved	Upper respiratory tract infections; herpes zoster virus infection	[220]
Baricitinib	I	JAK 1; JAK 2	Approved	Pulmonary and digestive infections	[221]
Upadacitinib	II	JAK 1	Approved	Sinus infections	[212]
Peficitinib	II	JAK 3; JAK 1	Phase 3	Lymphopenia	[222]
Filgotinib	II	JAK 1	Phase 3	Nasopharyngitis	[223]
Decernotinib	II	JAK 3	Phase 3	Cytopenias	[224]
Ruxolitinib	II	JAK 1; JAK 2	Phase 2	Anemia; headache	[219]
Itacitinib	II	JAK 1	Phase 2	Thrombocytopenia	[219]
